# Effect of a mental health training programme on Nigerian school pupils’ perceptions of mental illness

**DOI:** 10.1186/s13034-017-0157-4

**Published:** 2017-04-07

**Authors:** Adeola Oluwafunmilayo Oduguwa, Babatunde Adedokun, Olayinka Olusola Omigbodun

**Affiliations:** 1 Centre for Child and Adolescent Mental Health, University of Ibadan, Ibadan, Nigeria; 2 Department of Epidemiology and Medical Statistics, College of Medicine, University of Ibadan, Ibadan, Nigeria; 3 Department of Psychiatry, College of Medicine, University of Ibadan, Ibadan, Nigeria; 4 University College Hospital, Ibadan, Ibadan, Nigeria

**Keywords:** Effects, Mental health training programme, Mental illness, School children, Knowledge, Attitude, Social distance

## Abstract

**Background:**

Stigmatizing attitudes and discriminatory behaviour towards persons with mental illness are known to start in childhood. In Nigeria, it is not unusual to see children taunting persons with mental illness. This behaviour continues into adulthood as evidenced by the day-to-day occurrences in the community of negative attitudes and social distance from persons with mental illness. School-based interventions for pupils have been found to increase knowledge about mental illness. Children are recognised as potential agents of change bringing in new ways of thinking. This study determined the effect of a 3-day mental health training for school pupils in Southwest Nigeria, on the perceptions of and social distance towards persons with mental illness.

**Methods:**

A total of 205 school pupils drawn from two administrative wards were randomly assigned to control and experimental groups. The mean age of the pupils was 14.91 years (±1.3). The pupils in the intervention group received a 5-h mental health training session spaced out over 3-days. Apart from didactic lectures, case history presentations and discussions and role-play were part the training. Outcome measures were rated using a knowledge, attitude and social distance questionnaire at baseline, immediately following the training for both group and 3-week post intervention for the intervention group. A Student Evaluation Form was administered to evaluate the pupils’ assessment of the training programme. Frequencies, Chi square statistics, paired *t* test were used to analyse the data received.

**Results:**

At immediate post-intervention, the intervention group had a significantly higher mean knowledge score compared to controls, 21.1 vs. 22.0; p = 0.097 to 26.1 vs 22.0; p < 0.01. Respondents in the intervention group had a higher mean attitude score of 5.8 compared to 5.6 in the control group although this was not statistically significant (p < 0.627). Comparisons within the intervention group from baseline to immediate post-intervention showed a significant increase in mean knowledge and attitude scores of respondents, 21.0–26.2: p < 0.001 and 4.8–5.8; p = 0.004 respectively. This change was sustained at 3 weeks post intervention. The majority (98.8%) noted that the training was useful to them.

**Conclusions:**

Multiple contacts and mixed-method training sessions produced a positive and sustained change in knowledge of and attitude towards persons with mental illness in school pupils in Nigeria.

## Background

The burden of mental illness makes the need to create awareness and acceptance of affected persons in the populace more urgent [1]. Stigma and discrimination have been recognised as a major barrier to helping individuals with mental illness as well as their families [2].

Corrigan and colleagues identified protest, contact, and education as three major strategies for dealing with psychiatric stigma and discrimination [3].

Protest strategy is often described as a responsive approach that aims to challenge misrepresentations and negative beliefs about mental illness projected by the media and accepted by the public, but not necessarily replacing these unfavourable expressions with positive and factual information about mental illness [4]. Research has shown that anti-stigma strategies using protest have been effective but may have potential rebound effects [4–6].

Education strategy aims to provide factual information about mental illness and has been shown to improve the attitude of its target audience towards persons with mental illness, howbeit; the effects may not be sustained for a long period of time [7].

Contact strategy provides a platform for the public to meet and interact with persons with mental illnesses who are doing well on their jobs and are able to interact well with their neighbours [5, 8–10].

Most interventions aimed at improving the public’s perception of persons with mental illness have utilized one or more of these strategies while adjusting them to suit the target group. A meta-analysis of data from a total of 38,364 respondents recruited into 72 different studies which were conducted across 14 countries revealed that adolescents were more likely to be influenced by education strategy while adults were more like to be influenced by contact strategy [4]. There was no definitive report about the effect of protest strategy.

An uncontrolled intervention in selected secondary schools in the United Kingdom (UK) employed the use of contact and educational strategies to improve pupils’ perception of mental illness [10]. In the UK intervention, a total of 472 pupils received lectures, which included sessions delivered by a person who had experiences of living with mental illness [10]. At baseline, 1 week and 6 months follow-up, respondents completed a questionnaire that assessed their factual knowledge of, and attitude to mental illness on a Likert scale of “agree” “disagree” and “unsure”. Respondents’ desire for social distance was rated “definitely”, “probably”, “probably not”, “definitely not” and “not known”. Researchers reported significant changes across the three scales assessed at 1 week post intervention and these changes were sustained at 6 months follow-up [10].

Another study carried out in middle schools in the United States of America (USA) utilized educational strategies and incorporated activities such as games, poems, and storytelling [11]. At baseline, immediate post-intervention and 6 weeks follow-up, all respondents were required to complete questionnaires that assessed their knowledge of, and attitude towards persons with mental illness on a Likert scale of 5 from “strongly agree” to “strongly disagree”. Similarly, participants’ desire for social distance from persons with mental illness was measured on a Likert scale of 5 from “definitely unwilling” to “definitely willing”. Each of the questions on the knowledge, attitude and social distance scales were scored 1–5 based on the Likert scale and were such that higher scores on any of the 3 categories indicated accurate knowledge, positive attitude and favourable disposition towards persons with mental illness respectively. Responses from a total of 193 pupils were analysed; 87 in the control and 106 in the experimental groups. Findings from this study showed significant positive changes in the pupils’ mean knowledge, attitude and social distance scores at immediate post intervention [11]. These changes were sustained at 6 weeks post intervention.

A few intervention studies have also been carried out in developing countries. For instance, in rural Rawalpindi, a school mental health programme was developed to increase awareness about mental disorders and available treatment services [12]. The direct target group of the programme was school children who were required to share the information they were receiving with a parent, a neighbour, and a friend that did not attend the same school. The mental health programme incorporated activities such as lectures, short plays and skits, poster-paintings and essay writings [12]. Rahman and colleagues evaluated the impact of this school mental health programme on 50 school children aged 12–16 years in a rural sub-district of Rawalpindi who had been exposed to the programme for 4 months, and another 50 who did not receive the mental health training [12]. A 19-item questionnaire was used to assess mental health awareness of participants at baseline and 4 months post-intervention. Each item was rated on a scale of “yes”, “no” and “don’t know”, and for analysis, a score of “1” was assigned to every correct answer, “0” to incorrect and “don’t know” answers [12]. Researchers reported highly significant differences between the intervention and control groups such that schoolchildren who received the intervention, as well as their parents, neighbours, and friends all scored about five points higher than their counterparts in the control group [12]. Researchers also reported significant changes in the mean scores of school children in the control group and their friends, but this was minimal compared to the changes observed in the intervention group. The significant change among the control group was attributed to the fact that the questionnaire may have stirred up the desire to know more about mental health and thus personal enquiry into the subject matter [12].

Another intervention carried out among 78 secondary school pupils with a control group consisting of 76 students in Nigeria, utilised a single contact 3-h mental health training consisting of lectures and discussions [13].

Using an adapted questionnaire version of the UK Pinfold study, participants’ knowledge of, attitudes and social distance towards persons with mental illness were measured at baseline, immediate post-intervention, and at 6 months follow-up [13].

There were nine (9) knowledge and five (5) attitude items which were rated on a scale of “agree”, “disagree” and “not sure”, a score of 2 was given for each correct answer, 1 for “not sure” and 0 for the wrong response [13]. For the social distance scale, the five answer options were recoded into three by combining “definitely” and “probably” into a category and “definitely not” and “probably not” into another while “don’t know” was left as a separate category. Similar to the knowledge and attitude scales, a score of 2 was then assigned to correct responses, 1 for “don’t know” and 0 for a wrong response [13]. Researchers reported a significant increase in the mean knowledge score of participants in the study group compared to participants in the control group at immediate post intervention (11.4 vs. 9.5; p < 0.001), and this change was sustained at 6 months follow-up (11.3 vs. 9.3; p < 0.001) [13]. Researchers, however, suggested the need for intervention studies with longer duration and multiple training sessions to provide participants with more time to assimilate and internalise the training content; hence, resulting in a change in attitude and a reduction in the desire for social distance from persons with mental illness [13].

The use of role-play has been identified as an effective means of changing attitudes and challenging public views about stigmatising conditions such as HIV/AIDS [14]. It has also been found to achieve sustained positive behaviour and change, [15–17] but remains an unexplored intervention to improve perceptions of mental illness among school pupils in Nigeria. Therefore, the current study involved the conduct of a mental health training of three sessions over 5 h to challenge school pupils’ knowledge of mental illness, attitude and social distance towards persons with mental illness. The training programme included didactic lecture sessions, group discussions, and role play.

## Methods

### Study design

This was a quasi-experimental study with an intervention and a control group.

### Study setting

School pupils were recruited into the study as intervention and control groups from 2 wards selected from a list of 16 administrative wards that make up a district called *Ado*-*Odo Ota*, in Ogun state, Southwest Nigeria. The selected wards were a distance of 2 km apart to ensure that there was no contamination of participants in the control and intervention groups during the study. Two secondary schools were randomly selected from the control ward and three from the intervention ward, making a total of five schools. At the time of the study, the schools had no mental health syllabus in their curricula.

### Study participants

School pupils were selected in each Senior Secondary School 1 (SSS1) (Equivalent to 10 years of formal schooling) through to Senior Secondary School 3 (SS3) (Equivalent to 12 years of formal schooling) by randomly drawing numbers. Students picked from numbers written on small pieces of paper, mixed with papers that had no numbers, which were all neatly folded and shuffled. Only students who picked papers with numbers were recruited into the study.

### Study instruments

Measures were rated using an adapted version of the UK Pinfold questionnaire, which collects information about knowledge of mental illness, attitude towards, and desire for social distance from persons with mental illness [10], and had been adapted, translated and validated for use in Nigeria [13].

The terms, ‘mental health problems’ and ‘Schizophrenia’ in the questionnaire were replaced with ‘Mental illness’ and ‘Psychosis’ respectively. This was based on findings that ‘mental health problems’ and ‘Schizophrenia’ were confusing and strange terms to Nigerian pupils [13].

Four factual statements on post traumatic stress disorder (PTSD), psychosis, substance abuse, suicide, and self-harm were added to the adapted version based on the most common mental illnesses in Nigeria. There were 15 knowledge items in all, including statements such as, “One in four people will develop mental illness over the course of a lifetime”, “People can recover from mental illness”, “Bullying is a risk factor for suicide”, “People with post traumatic stress disorder often suffer from flashback and nightmares”. There were 8 attitude items including statements such as “People with mental illness are always difficult to talk to”, “People with mental illness are likely to become violent”, “People with mental illness are weak and have only themselves to blame”, “People with depression always like to be alone, feel sad & wish to die”. All knowledge and attitude items were rated on a Likert scale of “agree”, “disagree”, and “not sure”.

Four statements assessing social distance were rated “definitely”, “probably”, “probably not”, “definitely not” and “don’t know”. Sample questions include: “Would you feel afraid to talk to someone with mental illness?”, “Would you be upset to be in the same class with someone who had mental illness?”, “Would you be able to be friends with someone who had mental illness?”, “Would you be embarrassed if your friends knew that someone in your close family has a mental illness?”

Another questionnaire which was researcher-designed was used to collect information on participants’ evaluation of the mental health training programme. It consisted of open-ended statements and questions rated ‘yes’ or ‘no’. Sample of open-ended questions include “What did you like about the information you received?” “What did you not like about the information you received?”

## Procedure

**Fig. 1:**
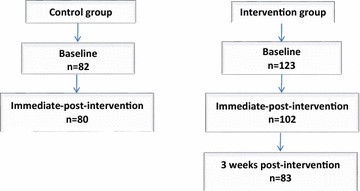
Outline of study procedure

Students in the intervention and control groups completed a questionnaire about their knowledge of mental illness, attitudes, and desire for social distance from persons with mental illness at baseline and immediate post intervention. At 3 weeks follow-up, the questionnaire was again administered to participants in the intervention group only, along with another questionnaire that assessed their evaluation of the intervention programme they received (see Fig. 1).

### The intervention

The intervention was a mental health awareness training delivered by A.O.O in a total of 5 h over 3 days; 2 h each on the first 2 days and 1 h on the third day. The content of the training manual was adapted from the “Training materials for multipurpose care workers in developing countries” [18], and the “Teachers’ knowledge, attitude & practice questionnaire” [19]. Both documents contained case vignettes that described the possible presentations, causes and treatment of mental illness which were adapted for the current study using teaching methods such as didactic lectures, group discussions, and role plays, to ensure students’ participation and learning.

On the first 2 days of the training, participants in the intervention group received didactic lectures that provided factual knowledge about mental health and illness. Participants also worked in groups of five that examined distressing behaviour and/or negative emotions presenting in the case vignettes, myths associated with mental illness, positive attitudes toward persons with mental illness and appropriate places to seek mental health care.

On the third and final day of the training, a recap of the major facts of the previous days’ training was done. Voluntary participants were selected to act a role play based on one of the case vignettes examined. At the end of the role play, major themes which the role play portrayed were discussed.

### Data analysis

Chi square test was used to compare the socio-demographic variables of participants in the control and intervention groups.

With the same scoring method used by Bella et al. [13], the fifteen knowledge and 8 attitude items on a Likert scale of “agree”, “disagree”, “not sure” were scored such that a score of 0 was assigned to every incorrect response, 1 for “not sure” responses, and 2 for correct responses. Therefore, total obtainable score on the knowledge items was 30 and 16 for the attitude items. Furthermore, the four social distance items, each on a Likert scale of 5, were recoded into 3 categories such that “definitely” and “probably” were merged into a category, “definitely not” and “probably not” were merged into another category, and “don’t know” was left as a distinct category [13]. As was done to the other scales, a score of 0 was assigned to responses that denoted unfavourable disposition, 1 for “don’t know” responses and 2 for responses that implied favourable disposition. The total obtainable score on the social distance items was therefore 8. This implied that the higher a participant’s score, the more favourable his or her disposition to persons with mental illness.

The mean knowledge, attitude and social distance scores were computed for both control and intervention groups at baseline and immediate post-intervention. Independent sample T test was used to compare means between the two groups at baseline and immediate post-intervention at a significance level of 5%. Furthermore, the general linear model was used to compare mean scores at baseline and immediate post intervention between both groups, while adjusting for age, gender and class. Adjusted mean differences in these scores between the intervention and control groups are reported with their 95% confidence intervals.

Repeated measures analysis of variance (rANOVA) was used to compare differences in the observed mean scores, with time as the within subject factor (three levels: baseline, immediate post- intervention, and 3 weeks follow-up). The Mauchly’s sphericity assumption was tested to ensure the equality of variance of the mean scores.

Students’ assessment of the training programme was presented in frequencies and percentages. Using thematic analysis, common themes in participants’ responses to the open-ended questions were grouped and also presented in frequencies and percentages.

## Results

### Sample characteristics

A total of 205 students were recruited at baseline; 123 in the intervention and 82 in the control groups. The total response rate at immediate post-intervention was 91.6% and at follow-up, the intervention group had a response rate of 66.7%. The high attrition rate at follow-up can be attributed to the uncertainties surrounding the election process scheduled in the country around the time of the study, and this resulted in the early vacation of schools. Participants in both control and intervention groups had similar socio-demographic characteristics (Table 1). Over half of the participants in each group were in the older age range; 15–17 years (62.8 and 56.6%). There were more females in the control group (51.9 vs. 43.8%) but this difference did not reach a statistical significance (p = 0.26) (see Table 1).

**Table 1:** Socio-demographic characteristics of the respondents

Variables	Intervention group frequency (%)	Control group frequency (%)	Total frequency (%)	Level of significance
Age group (years)				
10–14	45 (37.2)	36 (44.4)	81 (40.1)	x^2^ = 1.063
15–17	76 (62.8)	45 (56.6)	121 (59.9)	df = 1
Total	121 (100)	81 (100)	202 (100)	p = 0.303
Sex				
Male	68 (56.2)	39 (48.1)	107 (53.0)	x^2^ = 1.262
Female	53 (43.8)	42 (51.9)	95 (47.0)	df = 1
Total	121 (100)	81 (100)	202 (100)	p = 0.261
Class				
SS1	26 (21.5)	34 (42.0)	60 (29.7)	x^2^ = 5.911
SS2	55 (45.5)	25 (30.8)	80 (39.6)	df = 2
SS3	40 (33.0)	22 (27.2)	62 (30.7)	p = 0.052
Total	121 (100)	81 (100)	202 (100)	

### Effects of intervention

#### Between the intervention and the control groups

At baseline, mean knowledge scores of participants in the intervention and control groups were not significantly different (21.1 vs. 22.0; p = 0.097), however, at immediate post-intervention, participants in the intervention group had a mean score of 26.2, which was significantly higher than the mean score of 22.1 among the controls; p < 0.01.

There were no significant differences in the attitude and social distance mean scores of participants in both groups at baseline and at immediate post intervention (see Table 2).

**Table 2:** Comparison of knowledge, attitude and social distance scores at baseline and immediate post-intervention between intervention and control groups

	N	Mean scores (SD)	T	95% confidence interval	p
Baseline					
Knowledge scores					
Control	75	22.0 (3.9)	1.7	−0.2 to 2.0	0.097
Intervention	117	21.1 (3.5)			
Attitude scores					
Control	80	5.5 (2.0)	2.0	−0.2 to 1.1	0.058
Intervention	117	5.0 (2.1)			
Social distance scores					
Control	77	3.0 (2.3)	0.7	−0.9 to 0.4	0.485
Intervention	120	3.2 (2.1)			
Immediate post-intervention test					
Knowledge scores					
Control	74	22.1 (4.0)	7.4	−5.3 to −3.0	<0.01**
Intervention	101	26.2 (3.4)			
Attitude scores					
Control	79	5.6 (2.3)	0.5	−0.4 to 1.0	0.627
Intervention	108	5.8 (2.7)			
Social distance scores					
Control	78	3.0 (2.2)	1.1	−1.5 to 0.3	0.286
Intervention	108	3.3 (2.4)			

**p value significant at p < 0.05

Adjusting for age, gender and class, the mean knowledge score of respondents in the intervention group increased from 21.0 at baseline to 25.9 at immediate post-test, while participants in the control group had a mean score of 21.9 at baseline and 22.2 at immediate post-intervention and this difference in mean scores was significant (p < 0.05). Changes in mean attitude and social distance scores of participants in the intervention group were higher than those observed in the control group, but none of these differences reached statistical significance (see Table 3).

**Table 3:** General linear model comparison of knowledge, attitude and social distance scores at baseline and immediate post intervention between intervention and control groups

	Baseline	Immediate post	F	Adjusted mean difference*	95% CI p value*
All	Mean (SD)	Mean (SD)			
Knowledge score					
Intervention	21.0 (3.3)	25.9 (3.7)	*8.40*	*2.59*	2.01 to 3.16
Controls	21.9 (3.9)	22.2 (3.9)			<0.001**
Attitude score					
Intervention	4.9 (2.0)	5.8 (2.7)	1.22	0.48	0.02 to 0.95
Controls	5.5 (1.9)	5.6 (2.3)			0.27
Social distance score					
Intervention	3.1 (2.1)	3.4 (2.3)	0.75	2.10	−0.11 to 0.54
Controls	2.8 (1.6)	3.1 (2.2)			(0.39)

* Adjusted for age, gender, and class

**p value significant at p < 0.05

### Within the intervention group

There was a significant increase in participants’ mean knowledge score from 20.7 at baseline to 25.9 at immediate post-intervention but dropped slightly to 25.8 at follow-up (p < 0.01). There was also a steady increase in participants’ mean attitude scores from baseline to follow-up (4.9 to 5.8 to 6.0), and this was statistically significant (p = 0.02). Although there was a steady increase in mean social distance scores from baseline to follow-up (3.1 to 3.3 to 3.5), this change was not significant (p = 0.33) (see Table 4).

**Table 4:** Comparison of mean scores within the intervention group across the three-time points

	Baseline	Immediate post	3 weeks follow-up	Sphericity value	f	p value
All	Mean (SD)	Mean (SD)	Mean (SD)			
Knowledge score	20.7 (3.3)	25.9 (3.3)	25.8 (3.0)	0.82	83.55	<0.01**
Attitude score	4.9 (1.9)	5.8 (2.7)	6.0 (3.0)	0.97	4.22	0.02**
Social distance score	3.1 (2.2)	3.3 (2.5)	3.5 (2.4)	0.72	1.11	0.33

**p value significant at p < 0.05

### Effects on individual scale

In the intervention group, the percentage of participants who responded correctly, at immediate post-intervention, to knowledge items such as “Mental illnesses are caused by spiritual attack”, “One in four people will develop mental illness over the course of a lifetime”, and “Depression is a type of mental illness”, was significantly higher compared to baseline (p < 0.05). There was no difference on the items that stated that: “There is a stigma (shame) attached to people with mental health problems”, and “Parents with mental illness always transmit it to their children” (p = 0.08 and 0.36 respectively).

There was significant increase in the proportion of those who ticked “disagree” to attitude items such as “People with mental illness are always difficult to talk to” (12.4% at baseline to 29.6% at immediate post-intervention; p < 0.05) and “Psychosis is a spiritual problem that cannot be treated in the hospital” (43.5% at baseline to 74.1% at immediate post-intervention; p < 0.05).

The proportion of participants who responded favourably to the social distance item, “Would you feel afraid to talk to someone with mental illness?” was significantly higher at immediate post-intervention compared to baseline (22.2 vs. 38.9%; p < 0.05).

### What participants liked about the training programme

A total of 79 participants responded to the question, “What did you like about the information you received”. The majority of these participants (41.8%) reported that they liked the programme because it increased their awareness about mental illness, 19.0% liked the programme because it changed their belief about mental illness and 7.6% perceived that the programme helped them to develop empathy for people with mental illness (Table 5).

**Table 5:** Emerging themes from respondents’ answers on what they liked about the information they received

Generated themes and examples of students’ responses	n	%
Increased awareness about mental illness		
*“It makes you know more about the mental illness”*	33	41.8
*“To know the symptoms”*		
*“It educates me more about mental illness”*		
*“The information enlightened me on the issue of people with mental illness”*		
Increased ability to recognize someone with mental illness		
*“I would be able to identify a person with mental illness”*		
*“It makes me know the people with mental illness”*	4	5.4
*“It teaches us how to know people with mental illness”*		
Empathy		
*“It makes me understand the condition or the state of people with mental illness and how to relate with them”*		
*“The programme makes me understand my junior brother very well”*	6	7.6
*“I take pity on those who have mental illness”*		
Perceived change in belief or behaviour		
*“It makes me know that people with mental illness can be treated”*	15	19.0
*“that people with mental illness should not be tied down and flogged instead they should be taken to hospital”*		
*“I like the teaching about psychosis and mental illness because I thought that it was caused by a spiritual attack before”*		
Others	21	26.6
Structure/presentation/content of the lecture		
*“…I liked the way she classified the people disturbed with mental illness”*	17	81.0
*“The lecture was very interesting”*		
*“I liked the way they acted and demonstrated it”*		
Manner of relating		
*“I love how she speaks and demonstrates”*		
*“I like the lecturer’s teaching”*	4	19.0
*“She is good at explaining”*		

n > because of multiple responses

### What participants did not like about the training programme

There were responses from 24 participants with 20.8% stating that hearing about the symptoms of mental illness had created fear in them. Over half (54.2%) noted that they did not like the effects of the symptoms of mental illness on the persons affected and the behaviour of other people towards persons with mental illness (see Table 6).

**Table 6:** Emerging themes from respondents’ answers to what they did not like about the information they received

Generated themes and examples of students’ responses	n	%
Lecture methods		
*“I don’t like how they acted the drama”*	4	16.7
*“I didn’t like the group discussion”*		
Negative emotions		
*“When I was taught about the symptoms of mental illness, I was scared”*	5	20.8
Symptoms of/behaviour towards persons with mental illness		
*“I did not like the symptoms”*		
*“what I did not like is about the nightmares”*	13	54.2
*“about the situation of people with mental illness”*		
*“it is not good for someone to have mental illness”*		
Others		
*“…have not seen someone with mental illness”*		
*“…that mental illness is sometimes transmitted through stress”*	2	8.3

n > 24 because of multiple responses

### Participants’ evaluation of the training programme

The majority of the pupils affirmed that the programme was of benefit to them (92%), their school (71.1%), and their family (61.4). Most (48.2%) of the pupils noted that they learnt the most about mental illness from the lecture sessions and the least from the group discussions (3.6%). An equal proportion of students (38.6%) affirmed that they enjoyed the drama and lecture sessions the most.

Associations between participants’ evaluation of the training programme and their age and gender revealed that half (50%) of the females enjoyed the lecture sessions the most while more of the males (39.1%) enjoyed the drama sessions. Over half (52.8%) of the participants aged 10–14 years liked the drama sessions the most compared with 29.5% of the older (15–17 years) participants (p < 0.001) (see Table 7).

**Table 7:** Socio-demographic variables associated with participants’ response

Socio-demographic variables	N = 80			Difference
	What aspect of the training did you enjoy the most?			
	Lecture n (%)	Discussion n (%)	Drama n (%)	
Gender				
Male	15 (32.6)	13 (28.3)	18 (39.1)	x^2^ = 5.192
Female	17 (50.0)	3 (8.8)	14 (41.2)	p = 0.075
Age				
10–14	17 (47.2)	0 (0)	19 (52.8)	x^2^ = 16.616
15–17	15 (34.1)	16 (20.0)	13 (29.5)	p < 0.001**

Socio-demographic variables	N = 83			Difference
	From what aspect of the programme did you learn the most about mental illness?			
	Lecture n (%)	Discussion n (%)	Drama n (%)	
Gender				
Male	24 (51.1)	9 (19.1)	14 (29.8)	x^2^ = 0.755
Female	16 (44.4)	6 (16.7)	14 (38.9)	p = 0.685
Age				
10–14	17 (44.7)	6 (15.8)	15 (39.5)	x^2^ = 1.060
15–17	23 (51.1)	9 (20.0)	13 (28.9)	p = 0.589

**p value significant at p < 0.05

## Discussion

This study was conceived as a result of a recommendation from an earlier study on the impact of a mental health literacy training programme on Nigerian school children’s perception of mental illness and persons with mental illness. The study had achieved significant positive change in participants’ knowledge only, hence, the researchers suggested that subsequent studies should include more training sessions and multiple training methods in order to achieve significant improvement in participants’ attitude and desire for social distance [13]. Therefore, the mental health literacy programme in this study consisted of a 5-h training over a 3-day period, using lectures, discussion, and role-play teaching methods, as against the 1 day 3-h training that included lectures and discussions in the previous study [13]. However, this study did not include contact strategy because it was difficult to find persons willing to share their experiences with mental illness.

### Impact of the mental health training

Similar to the interventional study among Nigerian secondary school children in 2014 [13], this study achieved significant positive change in participants’ knowledge of mental illness. This positive change in knowledge is consistent with findings from other parts of the world [10, 12] and it corroborates the findings that educational strategies can cause positive changes in young people’s views of mental illness [10–13].

Comparisons between the control and intervention groups showed a positive change in the attitude of participants in the intervention group but this did not reach statistical significance. However, analysis within the intervention group revealed a significant positive change in participants’ attitude from baseline to immediate post-intervention, and a slight increase at follow-up. Differing opinions exist regarding the impact of role play on young peoples’ attitude towards persons with mental illness. A study among year 9 secondary school students in the UK, included role play and small group work in a workshop to increase participants’ mental health literacy and improve their attitude towards persons with mental illness [20]. Responses from participants showed positive changes in their perception of persons with mental illness, however, the study did not include a control group [20]. In another study among undergraduate students in the UK, researchers employed the use of role play only to improve participants’ attitude towards persons with mental illness. Although there was a positive change in the attitude of the participants, this did not reach statistical significance when compared with the control group [21].

A significant proportion of participants affirmed that they would not “feel afraid to talk to someone with mental illness” at post-intervention. Analysis of the overall items on the social distance scale, which measured perceived behaviour towards persons with mental illness, showed no significant change from baseline to post-intervention. Perceived behaviour is described as a person’s decision on what to do in a particular situation and it is often influenced by attitude and established norms, which are rooted in culture [22]. It is possible that the intervention delivered in this study had minimal influence on participants’ cultural beliefs, hence, the persistence of the desire for social distance. Less than 5% of the participants in the intervention group had stated that hearing about the symptoms of mental illness made them uncomfortable. Studies that have recorded changes in participants’ desire for social distance from persons with mental illness were for a longer duration, and incorporated contact strategy into the intervention programme such that participants interacted with persons who are successfully managing their mental illnesses [10, 23–25].

### Evaluation of the training programme

The majority of the participants indicated that they learnt the most from the didactic lectures. This may be because participants come from a formal school setting where the major method of teaching is the didactic lecture. It is worthy to note that participants least liked, and learned the least from the group discussion sessions. It may be that participants did not feel knowledgeable enough to discuss mental health and mental illness among themselves and may have been uncomfortable in the group discussions.

This study also attempted to measure participants’ perception of the impact of the training on their family and community using a self-report form for the participants. Although their responses were that their family and community benefitted positively from the training, it may not be entirely reliable. In a randomized trial conducted among school children in Pakistan to determine the impact of a school mental health programme, each study participant was asked to recruit a parent, a neighbour, and a friend who was not attending the same school into the study. Each study participant was also expected to teach his/her recruits what (s)he was learning from the training delivered [12]. The study reported significant improvement in the scores of all the participants at post-intervention, with the change most marked in the school children who participated in the mental health training and the least change in their neighbours.

The findings of this study reveal that there is a gap in secondary school children’s knowledge of mental illness, and attitude towards, and social distance from persons with mental illness. It also shows that secondary school children may respond positively to mental health training and that didactic teaching and role play, with multiple contact sessions are effective and acceptable methods of training among secondary school children.

## Limitations and strengths

This study is one of the few studies that achieved significant change in the attitude of participants using multiple teaching methods and sessions. The interval between the immediate post-assessment and follow-up was just 3 weeks and this period may not have been long enough to determine whether the effects of the training were sustained.

## Conclusions

Mental health training programmes with multiple training sessions and methods, delivered in schools, appear feasible for producing and sustaining positive change in school children’s knowledge of mental illness.

Mental health professionals need to partner with the Ministry of Education to develop a mental health syllabus for secondary schools that incorporates various participatory methods of learning and also provides a platform for students to meet and interact with persons with mental illness who have been able to successfully manage their illness and are living a good life.

Further research may be needed to ascertain the impact of role play on young persons’ perception of mental illness and persons with mental illness.

## Abbreviations

UK: United Kingdom
rANOVA: repeated measures analysis of variance
HIV/AIDS: Human Immunodeficiency Virus/Acquired Immune Deficiency Syndrome
